# A cross-sectional survey of supports for evidence-informed decision-making in healthcare organisations: a research protocol

**DOI:** 10.1186/s13012-014-0146-4

**Published:** 2014-10-09

**Authors:** Mathieu Ouimet, John N Lavis, Grégory Léon, Moriah E Ellen, Pierre-Olivier Bédard, Jeremy M Grimshaw, Marie-Pierre Gagnon

**Affiliations:** Centre Hospitalier Universitaire de Québec Research Centre, Québec City, Quebec Canada; Department of Political Science, Université Laval, Québec City, Quebec Canada; Centre for Health Economics and Policy Analysis, McMaster University, Hamilton, Ontario Canada; Department of Clinical Epidemiology and Biostatistics, McMaster University, Hamilton, Ontario Canada; McMaster Health Forum, McMaster University, Hamilton, Ontario Canada; Department of Political Science, McMaster University, Hamilton, Ontario Canada; Department of Global Health and Population, Harvard School of Public Health, Boston, Massachusetts USA; Jerusalem College of Technology, Jerusalem, Israel; Israeli Center for Technology Assessment in Health Care, Tel Hashomer, Israel; Department of Philosophy, School of Government and International Affairs, Durham University, Durham, UK; Clinical Epidemiology Program, Ottawa Hospital Research Institute, Ottawa, Ontario Canada; Department of Medicine, University of Ottawa, Ottawa, Ontario Canada; Faculty of Nursing Science, Université Laval, Québec City, Quebec Canada

**Keywords:** Health systems, Knowledge translation, Research evidence, Cross-sectional study

## Abstract

**Background:**

This protocol builds on the development of a) a framework that identified the various supports (i.e. positions, activities, interventions) that a healthcare organisation or health system can implement for evidence-informed decision-making (EIDM) and b) a qualitative study that showed the current mix of supports that some Canadian healthcare organisations have in place and the ones that are perceived to facilitate the use of research evidence in decision-making. Based on these findings, we developed a web survey to collect cross-sectional data about the specific supports that regional health authorities and hospitals in two Canadian provinces (Ontario and Quebec) have in place to facilitate EIDM.

**Methods/design:**

This paper describes the methods for a cross-sectional web survey among 32 regional health authorities and 253 hospitals in the provinces of Quebec and Ontario (Canada) to collect data on the current mix of organisational supports that these organisations have in place to facilitate evidence-informed decision-making. The data will be obtained through a two-step survey design: a 10-min survey among CEOs to identify key units and individuals in regard to our objectives (step 1) and a 20-min survey among managers of the key units identified in step 1 to collect information about the activities performed by their unit regarding the acquisition, assessment, adaptation and/or dissemination of research evidence in decision-making (step 2). The study will target three types of informants: CEOs, library/documentation centre managers and all other key managers whose unit is involved in the acquisition, assessment, adaptation/packaging and/or dissemination of research evidence in decision-making. We developed an innovative data collection system to increase the likelihood that only the best-informed respondent available answers each survey question. The reporting of the results will be done using descriptive statistics of supports by organisation type and by province.

**Discussion:**

This study will be the first to collect and report large-scale cross-sectional data on the current mix of supports health system organisations in the two most populous Canadian provinces have in place for evidence-informed decision-making. The study will also provide useful information to researchers on how to collect organisation-level data with reduced risk of self-reporting bias.

**Electronic supplementary material:**

The online version of this article (doi:10.1186/s13012-014-0146-4) contains supplementary material, which is available to authorized users.

## Background

Organisational absorptive capacity—the capacity for organisations to acquire and use external knowledge—depends on three key factors, namely, access to external sources of information (e.g. access to scientific journals), prior knowledge (e.g. employees’ skills in research methods) and social integration (e.g. ties with research producers and knowledge brokers) [1],[2]. An environmental scan in Canadian healthcare organisations and a scoping review of the literature on supports (i.e. positions, programs, interventions, instruments or tools) implemented across the health systems to support evidence-informed decision-making (EIDM) allowed us to identify four organisational-level components that can be found in an organisation’s research knowledge infrastructure: (1) climate for research use (e.g. mission, vision, values and strategic plan that reflect the value placed on the use of research evidence), (2) research production (e.g. ensuring that the appropriate research commissioning capacity is in place), (3) activities used to link research to action, which include push efforts (e.g. knowledge intelligence service that scans the research literature), facilitating pull efforts (e.g. enabling easy access to research evidence through physical tools and resources), pull efforts (e.g. training and continuing education that focus on finding and using research evidence in decision-making) and linkage and exchange efforts (e.g. meetings that highlight relevant research) and (4) evaluation efforts (e.g. monitoring and evaluation efforts on the use of research in decision-making) [3],[4]. In-depth semi-structured interviews conducted in three types of healthcare organisations (regional health authorities, hospitals and primary care practices) in two Canadian provinces (Ontario and Quebec) reveal the current mix of supports that these organisation have in place and the ones that are perceived to facilitate the use of research evidence in decision-making [3],[5]. Based on the findings of this qualitative study and of existing research syntheses [6]-[8], we developed a web survey to collect cross-sectional data about the specific supports that regional health authorities and hospitals in two Canadian provinces (Ontario and Quebec) have in place to facilitate EIDM.

The types of supports that will be examined in this study are those that have the potential to overcome the key barriers to research use, such as the lack of availability and of access to research (sub-optimal dissemination channels), poor clarity/relevance/reliability of research findings, poor timing/loss of opportunity, lack of user research skills and costs [6]. The types of supports that will be studied are also those that can be linked to the key facilitators of research use, such as availability and access to research (improved dissemination channels), improved clarity/relevance/reliability of research findings and research collaboration and ties between decision-makers, research staff and external researchers [6].

## Methods/design

The chosen research design is a cross-sectional web survey. Targeted participants are all regional health authorities and all general hospitals in Quebec and Ontario as categorised and reported by the web sites of their respective ministries of health. Specialised (non-general) hospitals, such as rehabilitation hospitals, hospitals for chronic patients and hospitals for psychiatric patients or patients suffering from addiction, will not be considered in this study. In Quebec, the study population includes 18 regional health authorities and 124 hospitals; in Ontario, the population includes 14 regional health authorities, namely local health integration networks (LHINs), and 129 hospitals.

**Figure 1:**
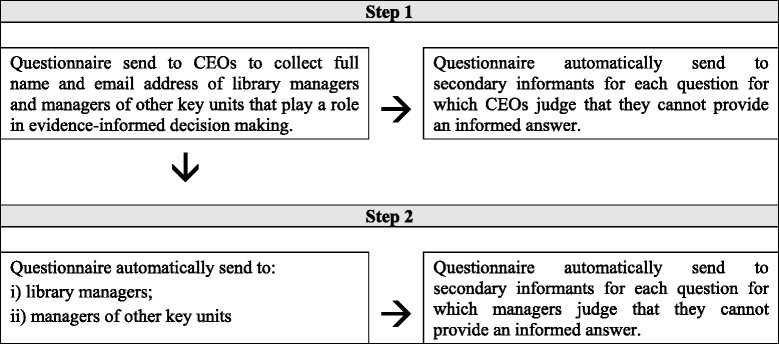
**Data collection process.**

The data will be collected through a two-step web survey design (Figure 1). In step 1, an invitation to participate in a 10-min survey will be sent by email to the CEO of each organisation to collect basic information about their organisation (e.g. mission, vision, values and accreditations) and to identify key units (e.g. in-house library or documentation centre, health technology assessment unit and clinical research and evaluation unit) and informants (e.g. managers of units that provide supports for evidence-informed decision-making). A letter that includes general information about the project will be sent to all CEOs by regular mail three weeks before sending the email invitation to participate that will provide them with the direct access to the web-based questionnaire.

In step 2, a 20-min survey that targets library/documentation units and other key units identified by the CEOs in step 1 will be conducted. Key informants will be the managers of these units that will have been identified by the CEOs in step 1. In step 2, the survey will examine three main supports of evidence-informed decision-making: research production, activities used to link research to actions—i.e. push, pull and linkage and exchange efforts—and evaluation efforts. The survey among library managers will include two additional questions pertaining to library services and subscription to academic journals/bibliographic databases (Table 1).

**Table 1:** **Themes covered in the survey by data collection (DC) step and type of informants**

Survey themes	Data collection step	CEO	Library manager	Manager of other units
Mention of importance of *research evidence* in formal mission, vision or values statements	1			
Certified by Accreditation Canada (hospitals only)	1			
In-house library or a documentation centre that provides access to research evidence	1			
Other units whose roles and activities might include acquiring, *assessing*, adapting/packaging and/or disseminating research evidence	1			
Direct access to a knowledge broker, i.e. someone whose role is primarily to keep the CEO informed about the latest research evidence that might be of strategic value	1			
Presence of a chief information officer	1			
Range of 12 services provided or not by the library to the organisation’s employees	2			
Refraining from subscribing to academic journals or bibliographic databases due to subscription costs	2			
Dissemination of research evidence to inform the decision-making process in one or more units	2			
Production of *systematic reviews*	2			
Production of traditional literature reviews or rapid reviews	2			
Production of *assessments* of the quality and local applicability of systematic reviews	2			
Production of summaries or abstracts of *primary studies*	2			
Production of summaries or abstracts of systematic reviews	2			
Organisation of interactive meetings or workshops to share new research evidence with staff	2			
Formal invitations to researchers from other organisations to share research evidence with members of the organisation	2			
Formal collaboration with researchers from other organisations in preparing primary studies	2			
Formal collaboration with researchers from other organisations in preparing systematic reviews	2			
Contracting out to provide training sessions within the organisation on how to acquire, assess, adapt/package, disseminate and/or use research evidence to inform decision-making	2			
Employees whose formal role includes acquiring, assessing, adapting/packaging, and/or disseminating research evidence	2			
Employees whose formal role includes establishing and/or maintaining relationships with researchers to inform decision-making	2			
Employees whose formal role includes the development of training tools aimed at increasing your organisation’s internal capacity to acquire, assess, adapt/package, and/or disseminate research evidence	2			
Resources and funding to monitor capacity to acquire, assess, adapt/package and/or disseminate research evidence	2			

Survey participants will be provided with a conceptual definition of all terms in italics.

Our team developed the survey questionnaires iteratively through multiple team meetings. The survey questionnaires solely include easy-to-answer, yes/no questions about organisational attributes. They do not include any question about individual attitude, belief or behaviour. Therefore, formal psychometric validation was not needed. Conceptual definitions of key terms such as `research evidence’ or `systematic reviews’ are provided in the questionnaires.

One of the main methodological concerns and challenges of surveys that aim at collecting organisation-level data is that they often rely on one or a few key informants that might not always be the most reliable informants to answer all survey questions. The challenge is thus to design a data collection procedure that will increase the likelihood that each survey question is being answered by the most informed respondent available.

In an effort to meet this challenge, we developed an innovative data collection procedure to increase the likelihood that only the best-informed respondent available answers each survey question. Survey participants who feel that they are not the right person to answer one or more questions will have the option not to respond and to provide the contact information of one or more persons (only one per unanswered question) from the same unit who he/she feels could provide a more accurate answer. The survey was programmed in a way that the one or more questions for which an informant reported not being the right person to provide an accurate answer will automatically be sent to the one or more secondary contact persons named by the primary informant. The secondary contact person(s) will then be invited to answer only the one or more questions that were allocated to him/her. Most importantly, the secondary contact person(s) will have the same option as the primary informant, that is, to forward one or more of these questions to a third informant, etc. This process will stop automatically after the fifth informant.

The entire web survey will be administrated by the services Centre APTI operated within the Faculty of the Social Sciences (Université Laval, Québec, Canada). The data collection will last for approximately five months. Up to three reminders will be sent to all survey respondents by email.

Data analysis will take the form of simple univariate and bivariate descriptive statistics of supports for evidence-informed decision-making. These supports will be cross-tabulated by type of organisation (i.e. regional health authorities vs. hospitals) and by province (i.e. Quebec vs. Ontario). We will also compute the proportion of survey questions for which primary informants identified secondary informants to minimise reporting bias.

### Ethics

The research protocol was submitted to the Ethics Research Committee of the CHU de Québec. The committee discussed the project in closed session on March 31, 2014. After evaluation and discussion, the committee noted that the research did not meet the definition of research involving human participants, according to the 2nd edition of *Tri-Council Policy Statement: Ethical Conduct for Research Involving Humans* (TCPS2 Article 2.1), given that the survey focuses on information that respondents are allowed to communicate as employees (the information relates to their work and does not target them personally). The Committee declared that the proposed project does not fall under the competency of the Committee. The McMaster University Research Ethic Board agreed with this decision.

Participation in this study will be entirely voluntary, and there will be no compensation provided for participation. Key informants will be able to put an end to their participation at any time without negative consequences or prejudice and without having to justify their decision. Proper arrangements will be made to ensure the confidentiality of the information provided by the key informants. The survey does not aim to evaluate the performance of the recruited organisations or to collect any information at the individual level. It will be impossible to identify individual informants or organisations in the publication or presentation of the study findings.

## Discussion

This study will be the first to collect and report large-scale cross-sectional data on the current mix of supports that health system organisations of the two most populous Canadian provinces have in place for evidence-informed decision-making. The study will also provide useful information to researchers on how to collect organisation-level data with reduced risk of self-reporting bias. Finally, the collected organisational data will be used to develop a protocol for a cross-sectional study aimed at examining organisational and individual correlates of research mobilisation by managers (and their advisers) of a random sample of the organisations that will participate to the organisational study described in this protocol.

## Authors’ original submitted files for images

Below are the links to the authors’ original submitted files for images. Authors’ original file for figure 1
