# Supplementary figures and images for: A cross-sectional survey of supports for evidence-informed decision-making in healthcare organisations: a research protocol
Source: Implement Sci. 2014 Oct 9;9:146. doi: 10.1186/s13012-014-0146-4 (PMC4197221; doi:10.1186/s13012-014-0146-4)

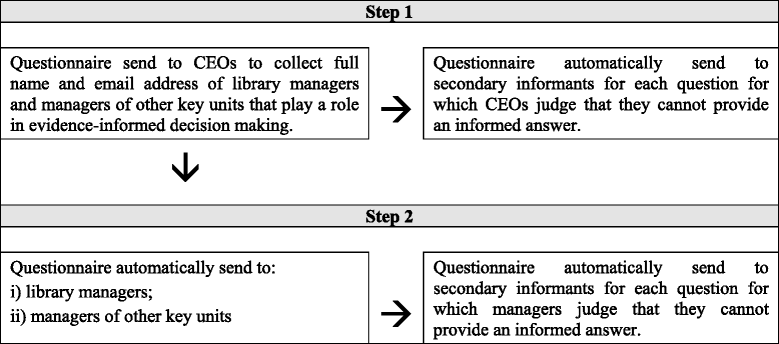

Supplement: Supplementary file 1 — Authors’ original file for figure 1 [file 13012_2014_146_MOESM1_ESM.gif]
